# Thymoma Exhibiting Spontaneous Regression With Cystic Change Due to Acute Infarction: A Case Report and Literature Review

**DOI:** 10.7759/cureus.56240

**Published:** 2024-03-15

**Authors:** Mikito Suzuki, Reiko Shimizu, Masahiko Harada, Tsunekazu Hishima, Hirotoshi Horio

**Affiliations:** 1 Thoracic Surgery, Tokyo Metropolitan Cancer and Infectious Diseases Center Komagome Hospital, Tokyo, JPN; 2 Pathology, Tokyo Metropolitan Cancer and Infectious Diseases Center Komagome Hospital, Tokyo, JPN

**Keywords:** thymoma, spontaneous regression, extended thymectomy, anti-acetylcholine receptor antibody, anterior mediastinal tumor

## Abstract

Spontaneous regression (SR) of thymoma is rare. We report a case of a surgically resected thymoma due to cystic changes owing to acute ischemic infarction with an increased anti-acetylcholine receptor antibody level.

A 61-year-old male underwent a computed tomography (CT) scan, which showed a 4.9 cm anterior mediastinal tumor and slight right pleural effusion. Blood test results indicated an elevated white blood cell count of 13300/mL. One month later, an enhanced CT scan at our hospital showed spontaneous mediastinal tumor regression to 3.7 cm and no pleural effusion. The tumor contained homogeneous low-density areas on enhanced CT, which showed high intensity on T2-weighted magnetic resonance imaging, indicating cystic changes. He had no symptoms of myasthenia; however, his anti-acetylcholine receptor antibody level was slightly elevated (0.4 nmol/L). Suspecting a thymoma, an extended total thymectomy through a median sternotomy was performed. Histopathological analysis confirmed the diagnosis of thymoma type B2 and Masaoka stage I. SR is due to acute intratumoral infarction. At two years postoperatively, no tumor recurrence or development of myasthenia gravis was observed.

Thymomas should be included in the differential diagnosis of anterior mediastinal tumors that regress spontaneously with cystic changes, pleural effusion, and an elevated inflammatory response. Mature cystic teratoma rupture should be differentiated, but preoperative biopsy is often challenging owing to necrotic and fibrous tissues; therefore, early surgical resection is required for diagnosis and treatment.

## Introduction

Thymoma is a rare thymic epithelial tumor, accounting for 0.2-1.5% of all malignant tumors [1]. Thymomas are typically slow-growing neoplasms. Thymomas regress with radiation, chemotherapy, and glucocorticoids [2], but rarely regress spontaneously [3-14]. We report a rare case of a patient with a surgically resected thymoma that had undergone spontaneous regression (SR) with cystic changes owing to acute intratumoral ischemic infarction.

## Case presentation

**Figure 1:**
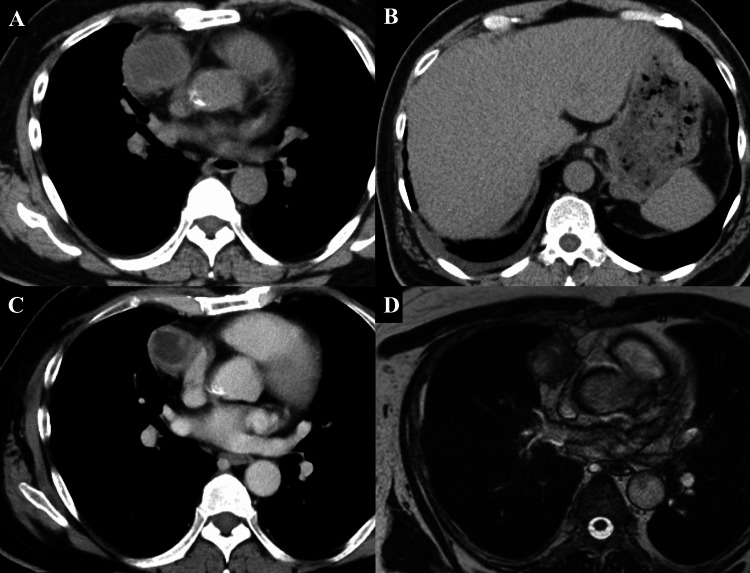
Preoperative radiological findings (A, B) Non-enhanced CT performed at a previous hospital showed a 4.9 cm anterior mediastinal tumor with fat stranding around the tumor and slight right pleural effusion. (C) One month following the preoperative hospital visit, enhanced CT showed that the anterior mediastinal tumor had spontaneously regressed to 3.7 cm. The fat stranding around the tumor and slight right pleural effusion had disappeared. (D) T2-weighted magnetic resonance imaging showed the mediastinal tumor contained a heterogeneous high intensity indicating a cystic change CT: computed tomography

A 61-year-old male underwent a computed tomography (CT) scan at a previous hospital as part of a medical check-up, which revealed a 4.9 × 4.9 × 4.2 cm anterior mediastinal tumor and slight right pleural effusion (Figure 1A, 1B). 

He reported no symptoms and had no abnormal vital signs. Blood test results showed an elevated white blood cell count of 13300/mL, and he was referred to our hospital for further examination and treatment. He was taking antidiabetic medication, statins, and proton pump inhibitors for diabetes mellitus, dyslipidemia, and gastroesophageal reflux disease, respectively.

**Figure 2:**
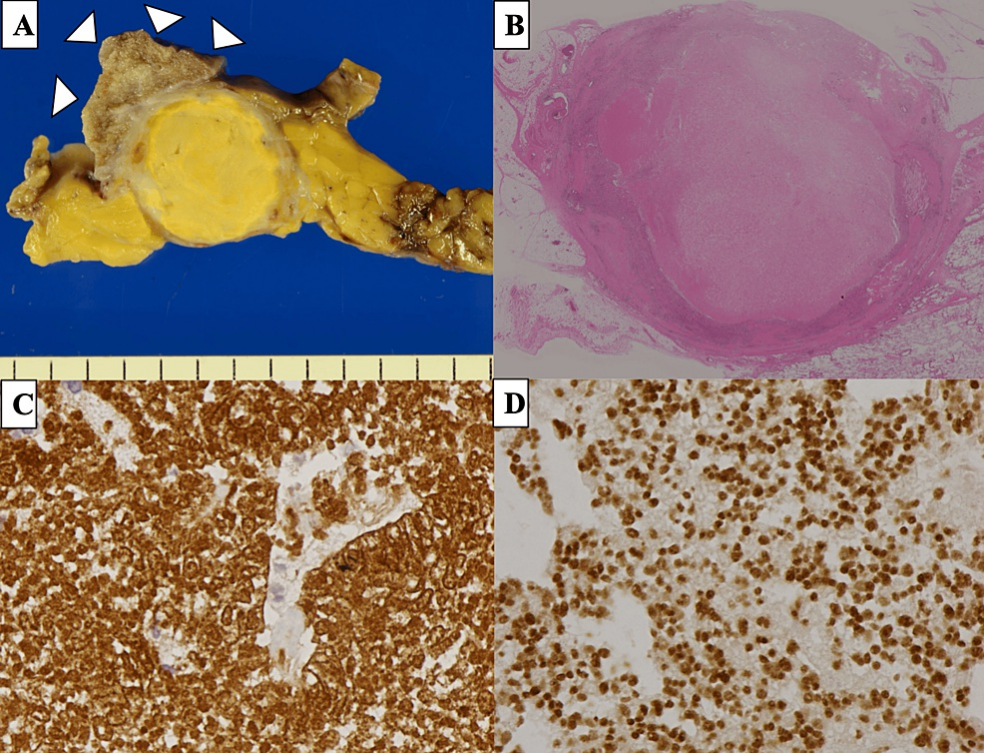
Pathological findings (A) The tumor is encapsulated with a broad yellowish inner area. The right middle lobe attached to the tumor was also resected (arrowheads). (B) The tumor contains a massive necrotic area in the center (hematoxylin-eosin staining). (C) The tumor included cytokeratin AE1/AE3-positive epithelial cells and TdT-positive immature lymphocytes (D), indicating type B2 thymoma TdT: terminal deoxynucleotidyl transferase

One month later, a chest-enhanced CT taken at our hospital showed that the anterior mediastinal tumor had spontaneously regressed to 3.7 × 3.5 × 3.1 cm (regression rate, 60%) (Figure 1C). Moreover, the right pleural effusion had disappeared. T2-weighted magnetic resonance imaging showed that the mediastinal tumor contained a low-intensity capsular area at the margin and a heterogeneous high-intensity area at the center, indicating cystic changes (Figure 1D). The patient's anti-acetylcholine receptor (AChR) antibody level was elevated at 0.4 nmol/L, but he had no myasthenia-related symptoms. The white blood cell count level had improved to 11700/mL. Masaoka stage I thymoma was suspected based on laboratory data and radiological findings. We performed an extended total thymectomy via median sternotomy. Intraoperatively, the tumor was found to be attached to the right middle lobe of the lung, and wedge resection of the right middle lobe was performed. No other adhesions were observed. The cut surface of the tumor showed a white cross-sectional outer surface and a broad, well-circumscribed, yellowish inner region (Figure 2A).

Pathologically, the tumor comprised prominent necrotic areas consistent with an inner yellowish area, with cholesterol clefts and hemosiderin deposits (Figure 2B) due to ischemic infarction. Immunohistochemically, a small number of tumor cells were AE1/AE3-positive (Figure 2C) with terminal deoxynucleotidyl transferase (TdT)-positive immature lymphocytes (Figure 2D), indicating type B2 thymoma. No microscopic lung invasion was observed; therefore, the patient was diagnosed with Masaoka stage I thymoma. Our patient had a good postoperative course and was discharged seven days after surgery. At two years postoperatively, no tumor recurrence or development of myasthenia gravis had been observed.

## Discussion

As the most common neoplasm of the anterior mediastinum, thymoma rarely regresses spontaneously [3-14]. The mechanisms of SR in thymomas include ischemic infarction and thromboembolism owing to sclerosing arteriopathy or occlusive disorders of the encapsulated tumor [3]. In this case, the encapsulated tumor mostly comprised necrotic tissue, in which the tumor cells partially remained. No obvious thromboembolism was detected; hence, we hypothesize that vascular occlusion due to rapid tumor enlargement or minute thromboembolism resulted in tumor necrosis.

English language studies published since 2000 in relation to thymomas exhibiting SR are summarized in Table 1 [4-14].

**Table 1:** Review of thymoma exhibiting SR in English language studies since 2000 *Elevated to 20 nmol/L during SR AChR: acetylcholine receptor; F: female; M: male; ND: not described; SR: spontaneous regression; WHO: World Health Organization; +: positive; -: negative

Authors	Country	Sex/age (years)	Anti-AChR antibody (nmol/L)	Pleural effusion	Tumor size (before/after SR, cm)	Duration of SR	Masaoka stage	WHO type
Okagawa et al. 2007 [4]	Japan	F/31	12	+ → -	6.0 × 5.5 × 5.0/4.5 × 4.0 × 3.0	3 weeks	II	B2
Hori et al. 2008 [5]	Japan	M/38	ND	-	3.5/2.8	1 month	II	B2
Yutaka et al. 2009[6]	Japan	M/47	ND	+ → -	8.0 × 7.0 × 6.0/6.0 × 3.0 × 2.0	1 month	III	B3
Nakazono et al. 2009 [7]	Japan	F/49	ND	-	ND/ND	2 weeks	I	B2
Nakazono et al. 2009 [7]	Japan	F/46	ND	+ → -	ND/ND	2 months	I	ND
Huang et al. 2009 [8]	Taiwan	F/52	-	-	2.5 × 2.0/0	3 years	ND	B2
Fukui et al. 2014 [9]	Japan	F/43	-	+ → -	3.4 × 3.4 × 2.7/1.4 × 1.2 × 0.8	1.5 months	II	B2
Fukui et al. 2014 [9]	Japan	F/32	ND	+ → -	10.0 × 9.5 × 7.9/7.3 × 6.9 × 4.9	1 week	IVa	B2
Toyokawa et al. 2014 [10]	Japan	M/28	ND	+ → -	11.8 × 5.8 × 2.7/10.0 × 3.8 × 1.0	3.5 weeks	I	B2
Furuya et al. 2015 [11]	Japan	M/30	-	+ → -	11.0 × 6.0 × 5.0/8.0 × 3.6 × 3.0	1 month	II	B2
Kikuchi et al. 2020 [12]	Japan	M/71	-	-	3.2 × 3.1/2.3 × 1.5	2 months	I	B3
Iijima et al. 2020 [13]	Japan	M/77	-	-	7.3 × 5.2 × 4.3/5.7 × 4.0 × 2.7	8 years	I	AB
Nishina et al. 2022 [14]	Japan	M/44	- → 20*	+ → -	5.5 × 4.3 × 3.4/3.0 × 3.0 × 1.8	4 years	II	B2
Our case	Japan	M/61	0.4	+ → -	4.9 × 4.9 × 4.2/3.7 × 3.5 × 3.1	1 month	I	B2

All studies were reported from Asia and mostly concerned Japanese patients. The median age of patients with thymoma who exhibited SR was 44 (range, 28-71) years, with a slightly higher male predominance [4,14]. The predominant histological subtypes are aggressive subtypes B2 and B3. Moreover, the median tumor size tends to be large (5.8 cm; range, 2.5-11.8 cm), with the Masaoka stage mostly being early stage [4,11,13,14]. The thick tumor capsule of such thymomas prevents invasion into the surrounding tissues, which is consistent with the present case. Inflammation secondary to ischemic infarction frequently causes chest pain and fever [3,4,11]. Pleural effusion also develops due to inflammation but mostly resorbs spontaneously with improvement in inflammation [4,6,7,9-11,14]. In this case, there was no pleural dissemination during surgery; therefore, the pleural effusion was considered to be derived from inflammation.

SR is often detected on follow-up CT scans during the waiting period from tumor detection to tumor biopsy or surgery. Prompt surgical resection is commonly performed to diagnose and treat anterior mediastinal tumors that are suspected to be thymomas. Therefore, the median observation period for SR is often reported to be as short as one month (range, one week to eight years) [4,11,13,14]. The high incidence of SR in Japan may be associated with the higher number of CT scanners than in other countries and the low threshold for repeated CT scans in a short period due to a national health insurance system. Further case studies are required to confirm this hypothesis.

When a mature cystic teratoma ruptures, the digestive enzymes produced by the pancreatic tissue within the tumor leak into the thoracic cavity and cause chest pain, fever, increased inflammatory response, and pleural effusion, such as in thymoma undergoing SR [15]. In addition, digestive enzymes from mature cystic teratomas are known to be at risk of severe adhesion to surrounding tissues such as the great vessels, lungs, and pericardium. Empyema and mediastinitis can also occur, worsening a patient's condition [15]. However, thymomas exhibiting SR have relatively light adhesions, often with only partial adhesion to the lungs [5,9-11,14]. Therefore, preoperative diagnosis and appropriate surgical strategies are important. A preoperative biopsy may be considered to confirm the diagnosis of anterior mediastinal tumors; however, this diagnosis is often challenging in thymomas exhibiting SR because of broad necrotic and fibrotic tissues, thereby limiting the indication for biopsy [4,8,10,11,14].

In our patient, increased anti-AChR antibody levels indicated a preoperative diagnosis of thymoma. Two previous studies have reported thymomas exhibiting SR with increased anti-AChR antibody levels [4,14]. Moreover, myasthenia gravis could develop during SR [14]. Cases of complete regression are rare [8], and surgery should be performed early for thymomas exhibiting SR and increased anti-AChR antibody levels, with consideration to the development of myasthenia gravis as in conventional cases.

## Conclusions

We encountered a rare case of a surgically resected thymoma exhibiting SR with cystic changes due to an intratumoral infarction. Thymoma should be considered a differential diagnosis in cases of spontaneously regressing anterior mediastinal tumors with an elevated inflammatory response and anti-AChR antibody levels and pleural effusion.
